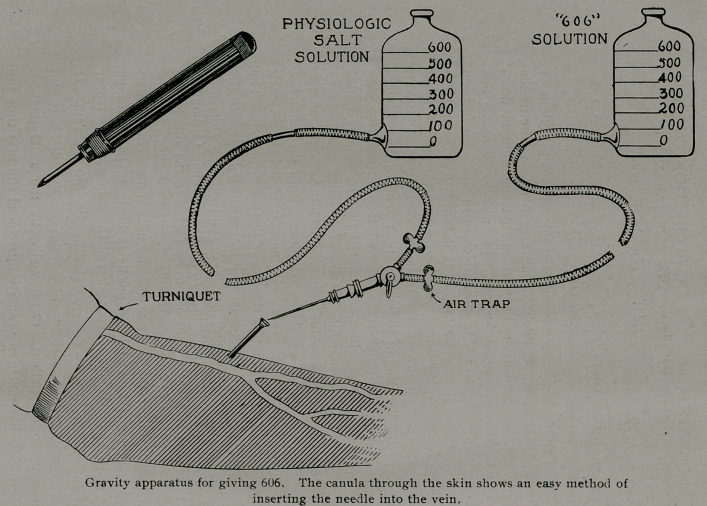# A Suggestion in the Introduction of the Needle into the Vein in Making Intravenous Injections of “606.”

**Published:** 1911-05

**Authors:** Edgar G. Ballenger, O. F. Elder

**Affiliations:** Atlanta, Ga.; Atlanta, Ga.


### A SUGGESTION IN THE INTRODUCTION OF THE NEEDLE INTO THE VEIN IN MAKING INTRAVENOUS INJECTIONS OF "606."

By Edgar G. Ballenger, M.D., and O. F. Elder, M.D., Atlanta, Ga.

All, I think, who have administered intravenous injections of "606," have, at times, experienced difficulty in passing the needle directly into the lumen of the vein so that the solution flows freely without a subcutaneous leak. In order to overcome this difficulty and to lessen the number of cases in which an incision through the skin is necessary or those in which a prolonged delay is caused by trying to pass the needle into the vein without the incision, the following technique is suggested: A trocar, about 12 inches long and of sufficient caliber to allow the introduction of the needle through it, is inserted just through the skin over the vein, after previous cocainization. The skin is pulled to one side of the vein while the trocar is being passed so as to avoid its entrance into the vein. If it has been passed too far through the skin it should be withdrawn until the point lies between the skin and the vein. A finger is now held on each side so as to keep the point of the trocar just over the vein. The needle connected by a three-way cock to the tubes leading to the physiologic salt solution and the "606" solution, as a rule, may be easily inserted into the vein through the canula. Ordinarily, the distended vein has a tendency to be forced to the side by the pressure required to carry the needle through the skin or if the vein is held under the point of the needle it (the vein) is flattened by the pressure and the needle is likely to pass through both walls of the vein. Having pierced the skin with the trocar little pressure is necessary to pass the needle into the vein. If not successful at first, subsequent effort causes very little pain and annoyance to the patient. Occasionally, by the usual method, if the needle has not been properly introduced the first time the escape of blood through the hole may produce a confusing swelling and necessitate a puncture at another point; if, however, the

Gravity apparatus for giving 606. The canula through the skin shows an easy method of inserting the needle into the vein.

trocar has been introduced previously, this blood is likely to escape through it without the formation of a hematoma. The writers have administered 199 injections of salvarsan, most of them being by the intravenous method; undoubtedly those which have been done after the previous injecton of the short trocar have been the most uniformly satisfactory. No matter what method is used the flow is always tested with physiologic salt solution, as elsewhere described, before "606" is allowed to flow in.

## Figures and Tables

**Figure f1:**